# Induction of Cell Death in the Human Acute Lymphoblastic Leukemia Cell Line Reh by Infection with Rotavirus Isolate Wt1-5

**DOI:** 10.3390/biomedicines8080242

**Published:** 2020-07-24

**Authors:** Rafael Guerrero, Carlos Guerrero, Orlando Acosta

**Affiliations:** Department of Physiological Sciences, Faculty of Medicine, Universidad Nacional de Colombia, Carrera 30 No. 45-03 Bloque 47, Ciudad Universitaria, Bogotá 111321, Colombia; raguerreror@unal.edu.co (R.G.); oacostal@unal.edu.co (O.A.)

**Keywords:** rotaviruses, oncolytic viruses, acute lymphoblastic leukemia, heat shock proteins, cancer treatment

## Abstract

Cancer is a major health problem that poses a great challenge to health care systems worldwide. Tools for cancer treatment have rapidly advanced in recent years, resulting in therapeutic strategies which are alternative and complementary to conventional treatment. To identify the cell surface receptors used by a tumor cell-adapted rotavirus and the cell death markers induced by its infection, we use Wt1-5, a rotavirus isolate recently adapted to tumor cells, to infect the human acute lymphoblastic leukemia cell line, Reh. The expression of cell surface receptors used by Wt1-5 was determined using flow cytometry and an antibody blocking assay to test for their implication in virus infection. Viral antigens and cell death markers induced by rotavirus infection were followed by flow cytometric analysis. The present study showed that rotavirus Wt1-5 was able to use cell surface proteins such as heat shock proteins (HSPs) 90, 70, 60 and 40, Hsc70, PDI and integrin β3. Rotavirus Wt1-5 induced cytotoxic effects including changes in cell membrane permeability, alteration of mitochondrial membrane potential, DNA fragmentation and activation of cell death signaling. Wt1-5 deserves to be further studied as a candidate oncolytic agent due to its ability to induce apoptosis in lymphoblastic leukemia-derived cells.

## 1. Introduction

Cancer is a major health problem that poses a great challenge to health care systems worldwide [1,2]. The steady growth of the older adult population is resulting in a greater incidence of cancer rates [3]. Acute lymphoblastic leukemia (ALL) has been defined as a malignant transformation and proliferation affecting lymphoid progenitor cells in the bone marrow, blood and extramedullary sites [4,5,6]. About 80% of ALL occurs in children, being the most common neoplasia among pediatric patients and producing a devastating disease in adults [4,5,6]. In high-income countries, the overall long-term survival rates for children affected with ALL are higher than 80%, while in low-income countries, these survival rates are much lower [5,7]. The pathogenesis characterizing ALL implicates abnormal cell proliferation and differentiation of a clonal population of lymphoid cells [8]. The conventional treatment of ALL consists of staged chemotherapy divided into induction, consolidation and long-term maintenance phases [9]. The induction therapy uses alternative protocols which include corticosteroids, vincristine, anthracycline cyclophosphamide, daunorubicin, methotrexate and cytarabine [4,5,9]. Consolidation and maintenance phases also include different protocols [4,5,9]. In general, chemotherapy is of high toxicity and has significant adverse effects [9,10], besides the resistance of malignant cells to therapy [11]. Although the control of ALL is achieved at a high percentage, relapsed or refractory ALL poses a challenge to conventional treatment. Standard chemotherapy has been combined with the use of monoclonal antibodies for targeting some leukemic surface antigens within the context of salvage therapies [12,13]. ALL cells express various cell surface antigens such as CD20, CD22, and CD19, which are amenable for targeted therapies. These leukemic surface antigens have been targeted by monoclonal antibodies, while decreasing toxicity to non-neoplastic cells [14]. New therapies, including tyrosine kinase inhibitors (TKIs), monoclonal antibodies, and CAR T-cell therapy, are changing the focus of therapies to treat recurrent or refractory cancers [15].

Research that aims at improving tools for cancer treatment has rapidly advanced in recent years. These advances have resulted in new alternative and complementary therapeutic strategies to conventional radiotherapy, chemotherapy, and surgery. Among these advances are microRNAs (miRNAs) [16], small interfering RNAs (siRNAs) [17,18], cell-based immunotherapy [19], inhibitors of signaling and metabolic pathways [20], targeting of cancer-related inflammation [21] and virotherapy [22,23]. Cancer cell-derived extracellular vesicles (EVs) have been used for systemic delivery of chemotherapeutic drugs and oncolytic viruses (OVs) [24]. A virus becomes a candidate to be used as an OV for effective treatment of cancer if it meets the following requirements: 1—selective replication within the tumor cells, 2—ability to spread within the tumor cells, 3—inability to infect normal cells and tissues, 4—lack of pathogenicity for humans, and 5—ideally appropriate to be genetically modified for improving its safety and efficacy [22,23]. The viruses currently used as oncolytic agents include adenoviruses, reovirus, herpes simplex virus (HSV), coxsackie virus (CV), vaccinia virus (VACV), measles virus (MV) and Newcastle disease virus (NDV) [25,26]. Some OVs have been used in combination with chemotherapeutic agents to improve the efficacy of cancer treatment [27].

Some characteristics of neoplastic cells make them susceptible to virus infection, while the virus tropism for tumor cells depends on the presence of cell surface receptors, cellular transcription machinery and signaling pathways [28,29]. The success of oncolytic virotherapy has been affected by the difficulties to overcome the limitations to deliver virus particles specifically and efficiently into the tumor cells [24,30]. One of the strategies to improve the specificity and efficacy of OVs involves the selection of tumor cells expressing cell surface receptors specific for virus entry into the target cells. Usually, these cell surface receptors are overexpressed in some tumor cells, but absent in normal cells, and are specific for a particular OV [28,31,32].

Oncolytic viruses (OVs) are ideal agents for the treatment of hematopoietic neoplasms because they can be systemically administered and use the specific expression of receptors in the target cells [33]. Recently, several OVs have been incorporated in clinical use. For instance, coxsackie virus A21 (CV-A21), an enterovirus, has shown a powerful cytostatic and cytocidal effect against three multiple myeloma (MM) cell lines, with reduced toxicity against normal peripheral blood mononuclear cells (PBMCs) [34]. The specificity of CV-A21 seems to be related to the expression of intracellular adhesion molecule-1 (ICAM-1) and cell surface decay-accelerating factor (DAF) [35]. Reovirus also offers a significant possibility for the treatment of hematological malignancies. The replication of reovirus is promoted by increased activation of Ras-signaling pathways [36,37], inducing cell death via necrotic, apoptotic, and autophagic pathways, in addition to improved immune response against tumor cells [38]. Reovirus does not affect normal PBMCs, although its clinical effectivity and spectrum in the treatment of hematological malignancies remains to be defined [39]. Within the rapid progress of new therapeutic strategies for the treatment of hematological malignancies, OVs continue to be a promising tool for effective treatments. Attempts to treat hematological malignancies with OVs have also included vesicular stomatitis virus (VSV) [40,41], live attenuated measles virus (MV) [42,43], oncolytic parvovirus [44], myxoma virus (MYXV) [45,46], and oncolytic poxvirus [47,48]. OVs have shown both advantages and limitations as therapy tools, including challenges and opportunities to improve treatment tolerability. OVs have been studied in myeloid leukemias and myeloma but not in precursor B-lymphoblastic leukemia, except in the case of treatment of pediatric B-lineage acute lymphoblastic leukemia in a preclinical context. These advances encourage additional research at preclinical and translational levels.

Rotavirus, a member of the family Reoviridae, is the most common etiological agent causative of severe gastroenteritis in children younger than five years of age worldwide. Rotavirus virions are composed of three concentric protein layers enveloping an 11-segmented dsRNA genome [49]. The entry of viruses into target cells shows a specific cell tropism involving the presence of receptor molecules on the cell surface. Entry of neuraminidase-sensitive rotaviruses into MA104 cells seems to be a multistep event that incorporates attachment to sialylated glycan followed by interactions with other cell surface molecules such as heat shock cognate protein 70 (Hsc70) [50,51,52], protein disulfide isomerase (PDI) [53,54,55] and integrins αVβ3 [56,57], α2β1 [58,59] and αIVβ1 [60,61]. Although rotavirus belongs to the same family of reovirus, both viruses show significant differences in cell tropism, receptors used for entry, particle structure, genomic segments, replication mechanisms, and clinical manifestations.

Some studies have reported an increased expression of heat shock proteins (HSPs) [62], PDI [63], and integrin αVβ3 [64] on the cell surface of tumor cells. In contrast, these proteins are only expressed at very low levels or not expressed in normal cells [65,66,67,68,69,70]. The role of these proteins has been associated with carcinogenesis through the regulation of cell proliferation, angiogenesis, cell migration, control of apoptotic pathways, resistance to cancer treatment, and metastasis in a wide range of cancers, including tumors from the brain, lung, prostate and breast, as well as in sarcomas and some lymphomas [71,72]. Hsp90, Hsp70, Hsc70, PDI and proteins αVβ3 have potential use as biomarkers for both diagnostic and prognostic purposes, and also as therapeutic targets for cancer treatment [64,71,72].

In a recent work, it has been shown that rotaviruses can be adapted to successfully infect tumor cells that express HSPs, PDI and integrin β3 on their outer cytoplasmic membrane [73]. Taking this previous background research into account, we investigated in the present work the potential utility of the tumor adapted rotavirus isolate Wt1-5 to infect, replicate and induce cell death in the human acute lymphoblastic leukemia cell line Reh. We found that rotavirus isolate Wt1-5 was able to produce cytotoxic effects in Reh cells that were represented by changes in the cell membrane integrity, mitochondrial membrane potential, DNA fragmentation, and activation of cell death signaling. Strikingly, cell surface proteins Hsp90, Hsp70, Hcs70, αVβ3, and PDI were found to participate during rotavirus entry into the Reh cells. The present results support the idea that rotavirus isolate Wt1-5 can be an interesting potential candidate to be further studied in the context of oncolytic agents in vitro assays involving human acute lymphoblastic leukemia cells.

## 2. Materials and Methods

### 2.1. Cells

Reh, a human acute lymphoblastic leukemia cell line (ATTC^®^ CRL-8286™), was kindly donated by Dr. J. P. Vernot (Faculty of Medicine, Universidad Nacional de Colombia). Cells were cultured in T-25 flasks (Corning^®^, Elmira, NY, USA) in RPMI 1640 (Sigma-Aldrich^®^, St. Louis, MO, USA) supplemented with 10% fetal bovine serum (FBS) (Gibco ^®^, Gaithersburg, MA, USA), 20 U/mL penicillin and 40U/mL streptomycin, and incubated in a humidified atmosphere with 5% CO_2_ at 37 °C.

### 2.2. Isolation of PBMCs

The study protocol involving volunteers was approved by the Ethics Committee of the Faculty of Medicine of Universidad Nacional de Colombia, and written informed consent was obtained. Peripheral blood mononuclear cells (PBMCs) were collected by venipuncture from 8 healthy volunteers, using heparinized BD Vacutainer^®^ ™ (REF 362753, Franklin lakes, NJ, USA) containing sodium heparin solution as an anticoagulant, polyester gel, and FICOLL ™ Hypaque ™ solution. Blood samples were centrifuged at 800× *g* for 20 min at 18–20 °C, and the supernatant plasma was removed. The layer containing the mononuclear cells was transferred to a conical polystyrene tube. Blood samples were centrifuged at 800× *g* for 20 min at 18–20 °C, and the supernatant plasma was removed. The layer containing the mononuclear cells was transferred to a conical polystyrene tube. PBMCs were washed by two cycles of addition of PBS and centrifugation at 400× *g* for 10 min at 18–20 °C, and then, were resuspended in RPMI medium. Counting of cells was performed using a Neubauer chamber. Cell viability was determined using the trypan blue exclusion test.

### 2.3. Virus

The rotavirus isolate Wt1-5 was described elsewhere by Guerrero et al., 2016 [73]. Briefly, rotavirus Wt1-5 was obtained through adaptation to tumor cells of a combination of five rotavirus isolates (Wt1 to Wt5) purified from the fecal samples of children. Rotavirus Wt1-5 was cesium chloride gradient-purified from infected Reh cells and stored in TMS buffer (10 mM Tris-HCl, pH 7.4, 150 mM NaCl and 1 mM MgCl_2_). Virus particles were activated with trypsin (1 μg/mL) (Sigma-Aldrich) at 37 °C for 30 min [74].

### 2.4. Virus Titration

Stocks of the purified virus were diluted serially in RPMI 1640 without FBS and used to inoculate Reh cells (2 × 10^6^ cells/well) in 48-well culture plates. The viral inoculum was removed after 1 h incubation at 37 °C and the cells were further incubated for 11 h in fresh medium before fixation in 4% paraformaldehyde (PFD) in PBS. Cells positive to rotavirus antigen were detected in an immunocytochemistry assay, as described below, to determine the virus titer in terms of focus forming units per mL (FFU/mL) [74].

### 2.5. Inactivation of Rotavirus

Rotavirus isolate Wt1-5 (10 mL; 1.2 × 10^10^ FFU/mL) was inactivated in a laminar flow cabinet by exposure to UV light (254 nm; 720 µW/cm^2^; Hoefer UVC 500 Ultraviolet Crosslinker) at a distance of 5 cm for 60 min at room temperature to crosslink its RNA. The inactivated virus particles were used as a control in the cell viability and cell signaling activation assays [75,76].

### 2.6. Cell Infection

Reh cells were washed twice in RPMI 1640 without FBS, and then, seeded (2.0 × 10^5^ cells/well in 300 mL) in a 48-well culture plate. The cells were inoculated with rotavirus at different MOIs (0.01, 0.05, 0.1 0.5, 1, 2, 3, and 6) of trypsin-activated rotavirus Wt1-5 in FBS-free medium and incubated at 37 °C for 30 min. Non-infected cells and cells infected with UV-inactivated rotavirus and Wt1-5-infected PBMCs at the same MOIs were used as a control. Cells were harvested at 24 h.p.i. and fixed in 4% PFD in PBS for 30 min at room temperature (RT) before two washes in PBS. Cells were resuspended in PBS before processing for immunochemistry assay [77,78].

### 2.7. Reinfection Assay

To approach the rotavirus cell cycle, Reh cells and PBCs were inoculated with rotavirus isolate Wt1-5 (MOI 1 and 2) as indicated above. Cell aliquots were collected at 2, 4, 6, 8, 10, 12, 24, 36 and 48 h.p.i., and then, frozen and thawed twice before being centrifuged at 700× *g*. Infectious virion production was assayed by reinfecting new Reh cells with the 700 g supernatant previously activated with trypsin. Infection level was determined at 12 h.p.i. by detecting the rotavirus antigen-containing cells using an immunochemistry assay as described below.

### 2.8. Immunocytochemistry Assay

Reh cell and Wt1-5-infected PBMCs suspensions were applied onto coverslips, permeabilized with 0.5% Triton X-100 for 5 min at RT, fixed with PFD, and then, washed twice with PBS for 5 min each time. The cells were treated with rabbit hyperimmune serum (1:2000) against rotavirus structural proteins, tested by Western blotting for the absence of antibodies to Reh cell proteins. Serum-treated cells were incubated for 1 h at 37 °C and washed twice with PBS before the addition of donkey anti-rabbit secondary antibody conjugated to horseradish peroxidase (HRP, 0.13 mg/mL, Santa Cruz Biotechnology Inc., SC-2313, Paso Robles, CA, USA) and incubation for 1 h at 37 °C. The cells were washed twice with PBS and the HRP activity revealed with AEC (3-Amino-9-ethylcarbazole) in acetate buffer (30 mM sodium acetate, 12 mM acetic acid, pH 5.0) containing 0.36% hydrogen peroxide. The percentages of rotavirus antigen-positive cells were recorded using the software ImageJ and 10 representative photographs of each coverslip were taken [78].

### 2.9. Flow Cytometry

Reh cells and PBMCs were infected with rotavirus isolate Wt1-5 (MOI 1 or 2) as indicated above. The cells at 24 h.p.i were fixed with 4% PFD in PBS at RT for 30 min, washed twice with PBS and permeabilized with 0.5% Triton X-100 for 5 min at RT. The cells were treated with NH_4_Cl and 0.1 mM glycine in PBS for 30 min at RT for diminishing cell’s autofluorescence signals. PBS-washed cells were reacted for 1 h at 37 °C with rabbit hyperimmune serum (1:2000 in PBS containing 1% BSA) against rotavirus structural proteins and then washed twice with PBS before addition of goat FITC-conjugated anti-rabbit IgG (0.88 mg/mL, Santa Cruz Biotechnology Inc., SC-2024, Paso Robles, CA, USA) diluted in PBS containing 1% BSA and incubation for 30 min at RT in the dark. Samples were analyzed on a BD FACSCanto™ II Flow Cytometer using the FlowJo software version 10.6 or FACSDiva™ software version 8.0 (Becton-Dickinson, Franklin lakes, NJ, USA). Isotype antibodies were used as negative control. Median fluorescence intensity (MFI) values were determined for both positive and negative cell samples.

### 2.10. ELISA

ELISA plates with 96 wells were coated by incubation for 1 h at 37 °C with antibodies from guinea pig hyperimmune serum (1:1000 diluted in PBS containing 1% BSA) against rotavirus structural proteins, and then, washed three times with washing buffer (PBS with 0.05% Tween 20, PBS-T). Non-specific binding sites were blocked by incubating with 5% skimmed milk in PBS-T for 1 h at 37 °C, followed by washing twice with PBS-T and addition of 700 g supernatant samples (in triplicate) from rotavirus Wt1-5-infected Reh cells and Wt1-5-infected PBMCs at 24 h.p.i., as indicated above. Supernatants from Reh cells infected with UV-inactivated rotavirus Wt1-5 and supernatants from cells where no virus was added were used as a negative control. The supernatants were incubated overnight at 4 °C, and then, the wells were washed three times with PBS-T before the addition of rabbit hyperimmune serum (1:2000 diluted in PBS containing 1% BSA) against rotavirus structural proteins. After incubation for 1 h at 37 °C and washing twice with PBS-T, secondary HRP-conjugated goat anti-rabbit IgG antibodies (0.13 μg/mL in PBS containing 1% BSA, Santa Cruz Biotechnology Inc., SC-2301) were added and incubated for 1 h at 37 °C. Following washing twice with PBS-T, the reaction was developed using o-phenylenediamine (OPD) and H_2_O_2_, and the reaction stopped by adding 2 N H_2_SO_4_. Plates were read in an Ultramark™ Microplate Reader, model 8422 (Bio-Rad). The net value of optical density (OD) at 490 nm was obtained after subtraction of the negative control value and values were expressed as ΔOD [78].

### 2.11. Cell Viability

Reh cells and PBMCs were inoculated with rotavirus isolate Wt1-5 at the MOIs indicated above. The permeability of the cytoplasmic membrane was assessed using the trypan blue exclusion test. Cells in RPMI culture medium were mixed with an equal volume of 0.4% trypan blue in PBS for 1 min at RT. Cells in a Neubauer chamber were observed using an inverted microscope (Euromex). Non-infected or H_2_O_2_-treated cells were used as the control. Cell viability was expressed as the percentage of viable cells compared with non-infected control cells referred to as 100%.

### 2.12. Proliferation Analysis

The Reh cells and PBMCs were collected by centrifugation at 400× *g* for 5 min and the supernatant carefully discarded. The cells were washed twice with RMPI 1640 without FBS and resuspended (1 × 10^6^ cells/mL) in working solution of CellTracker™ Blue CMAC Dye (Life Technologies, Thermo Fisher Inc., Rockford, IL, USA) prepared in RPMI 1640 without FBS at a concentration of 0.5 mg/mL at 37 °C. The cells were incubated for 30 min at 37 °C in a 5% CO_2_ atmosphere and then centrifuged at 400× *g* for 5 min to remove the unbound dye. The cells were resuspended in RPMI 1640 at 1 × 10^5^ cells/mL without FBS, seeded (300 mL/well) in a 48-well plate and inoculated with trypsin-activated rotavirus Wt1-5 (MOIs of 1, 2, 3 or 6). The infected cells were incubated at 37 °C and harvested at the indicated post-infection times. The harvested cells were analyzed using a BioTek FLx800 Microplate Fluorescence Reader with a 370 nm excitation filter and a 470 nm emission filter. The intensity of fluorescence was expressed in arbitrary units of fluorescence (AUF). Representative photographs of each treatment were taken using a fluorescence microscope (VanGuard). Control cells consisted of non-infected cells, H_2_O_2_-treated cells, and cells inoculated with UV-inactivated rotavirus Wt1-5 at the same MOIs. 

### 2.13. Cell Lysis Assessment

Reh cells and PBMCs that had been infected with rotavirus Wt1-5 at MOIs of 1, 2, 3 or 6 and cultured in RPMI culture medium without FBS were harvested at 0, 24, 32 y 48 h.p.i. and centrifuged at 400× *g* for 5 min. The collected supernatants were tested for the lactate dehydrogenase (LDH) activity using a commercially available LDH kit (BioSystem, Abcam, Cambridge, MA, USA) following the manufacturer’s instructions. Samples were recorded at 340 nm using a GENESYS™ 20 spectrophotometer (Thermo scientific Inc., Rockford, IL, USA). Non-infected cells, H_2_O_2_-treated cells, and cells inoculated with UV-inactivated virus at MOI of 2 were used as a control. 

### 2.14. Cytotoxic Effect Assay

Reh cells and PBMCs were inoculated with rotavirus Wt1-5 at increasing MOIs (0.05 to 6) and cultured in RPMI 1640 without FBS. After the point times were reached (12 and 24 h.p.i.), the medium was removed after centrifugation at 400× *g* for 5 min and the cells washed twice with PBS. The cells were seeded (100 μL) at a density of 6.6 × 10^5^ cells/mL in 96-well plates in RPMI 1640 without FBS and containing 0.4 μM resazurin (Amresco^®^ Code 0695-25G, Solon, OH, USA). The cells were incubated for the indicated times at 37 °C and in a 5% CO_2_ chamber and the plates were read with a 536 nm excitation wavelength and a 595 nm emission filter using a BioTek Cytation 3 Cell Imaging Multi-Mode Reader (BioTek Winooski, VT, USA). Control cells were as described in the above subsection.

### 2.15. ∆Ψm Assay

Rotavirus Wt1-5-inoculated Reh cells and PBMCs at a density of 1 × 10^6^ cells/mL and at 24 h.p.i. were incubated with 20 nM DiOC6(3) (3,3′-dihexyloxacarbocyanine iodide, Life Technologies, Cat: D-273, Thermo Fisher Inc., Rockford, IL, USA) and 2 μg/mL (1.6 μM) 7-AAD (7-Aminoactinomycin D, Life Technologies, Cat: A1310, Thermo Fisher Inc., Rockford, IL, USA) in PBS for 15 min at 37 °C, protected from the light. DiOC6(3) is a membrane-permeable lipophilic fluorochrome to detect mitochondrial transmembrane potential (∆Ψm) [79,80,81]. A low signal of DiOC6(3) indicates a collapse of ∆Ψm. Dyes were removed by centrifugation at 400× *g* for 5 min at RT and washed twice with PBS. The cells resuspended in RMPI 1640 without FBS were immediately analyzed by flow cytometry using a BD FACSCanto II Flow Cytometer. Data were analyzed using BD FACSDiva 6 or FlowJo software version vX.0.7 (Becton-Dickinson, Franklin lakes, NJ, USA). Dead cells were excluded from the analysis according to their 7-AAD spectral emission. Non-infected cells in the absence of FBS and cells inoculated with UV-inactivated rotavirus Wt1-5 were used as a control.

### 2.16. Annexin V and 7-AAD Assay

Reh cells and PBMCs were inoculated with Wt1-5 (MOI 2) or UV-inactivated Wt1-5 (MOI 2) and cultured in RPMI medium without FBS. The cells were incubated at 37 °C and harvested at 24 h.p.i. Reh cells without virus inoculation were used as a control. The cells were collected by centrifugation at 400× *g* for 5 min and washed twice with sterile PBS at RT. The cells were resuspended at a density of 1 × 10^6^ cells/mL in 100 μL of annexin V binding buffer containing 10 μM of annexin V (Annexin V-Alexa Fluor™ 488 conjugate. Thermo Fisher Cat: A13201, Rockford, IL, US), 1.6 μM 7-ADD (7-Aminoactinomycin D, Life Technologies, Cat: A1310, Thermo Fisher, Rockford, IL, USA), 2 μM Hoechst 33342 (Thermo Fisher. Cat: H1399, Rockford, IL, USA), 10 mM HEPES, pH 7.4, 140 mM NaCl, and 2.5 mM CaCl_2_. The cells were incubated for 20 min at RT in the dark. The cells (1 × 10^4^ in 20 μL) were placed onto a glass slide, covered with a coverslip and kept on ice before being analyzed in a Nikon C1 confocal laser scanning microscope controlled by the software EZ-C1 for Nikon, Gold version 3.90, using the smallest pinhole (30 mm) (Nikon Instruments, Tokio, Japan). Four representative fluorescent images were obtained using sequentially neon helium laser (568 nm, red) and argon laser (488 nm, green; 408 nm, blue). Reh cells treated with 20 nM doxorubicin for 24 h were used as cell death control, whereas non-infected Reh cells were used as cell viability control.

### 2.17. Caspase Assay

Caspase 3 and 8 activities in samples from rotavirus-infected Reh cells were measured using the Vybrant^®^ FAM Caspase-3 and -7 Assay Kit (Life Technologies. V35118, Thermo Fisher, Rockford, IL, USA), and Vybrant^®^ FAM Caspase-8 Assay Kit (Life Technologies. V35119, Thermo Fisher, Rockford, IL, USA), respectively. Samples from Reh cells infected with rotavirus Wt1-5 (MOIs of 1 and 2) were prepared by collecting cells from the wells of 48-well plates at 24 h.p.i. and centrifuging them at 400× *g* for 5 min. PBS-washed cells (1 × 10^6^ cells/mL) in 300 mL RPMI culture medium were transferred into flow cytometry tubes and immediately added with 10 mL of 30X FLICA (Fluorescent-labeled inhibitor of caspases) and incubated for 1 h at 37 °C and 5% CO_2_ in the dark. The cells were washed twice, resuspended in 1 mL of washing buffer and collected by centrifugation at 400× *g* for 5 min. The supernatant was discarded and cells were resuspended in washing buffer. The fluorescence intensity (488 nm excitation wavelength; 564 nm emission wavelength) was measured using a BD FACS Aria II flow cytometer. Data analysis was conducted using BD FACSDiva 6 or FlowJo software version vX.0.7 (Becton-Dickinson, Franklin lakes, NJ, USA). Non-infected cells and cells infected with UV-inactivated virus were used as a control.

### 2.18. TUNEL Assay

DNA fragmentation following rotavirus Wt1-5 infection was assessed using an In Situ Cell Death Detection Kit, POD (TUNEL, Roche Cat. No. 11 684 817 910, Branchburg, NJ, USA). Reh cells were infected with rotavirus Wt1-5 (MOIs of 0.1 to 6) as indicated above and cultured in 48-well plates in RPMI without FBS. The cells were harvested at 24 h.p.i., collected by centrifugation at 400× *g* for 5 min and fixed with 4% PFD in PBS at RT for 1 h. The cells were newly collected by centrifugation, washed twice with PBS and applied onto coverslips. The cells were treated with blocking solution (3% H_2_O_2_ in methanol) and washed twice with PBS before permeabilization with 0.5% Triton X-100 for 5 min on ice. After two washes in PBS, the cells were incubated in the dark for 1 h at 37 °C with 3 mL of the DNA labeling solution containing terminal deoxynucleotidyl transferase and fluorescein-dUTP in reaction buffer. Fixed and permeabilized cells incubated in labeling solution without the enzyme were used as a negative control, while fixed and permeabilized cells incubated for 10 min at RT with DNase I grade I, RNase-free (Thermo Fisher™ Catalog number: EN0521, Rockford, IL, USA) (3 U/mL in 50 mM Tris-HCl, pH 7.5, 10 mM MgCl_2_ and 1% BSA) were used as a positive control. Non-infected cells and H_2_O_2_-treated cells were also used as control. The cells were simultaneously assessed for the presence of structural rotavirus antigens and nuclei by incubating cells with rabbit hyperimmune serum against viral antigens for 1 h at 37 °C, followed by washing twice with PBS and addition of secondary goat anti-rabbit Alexa Fluor 568-conjugated IgG antibody (Santa Cruz Biotechnology Inc., SC-2780, Paso Robles, CA, USA) in PBS containing 1% BSA. The cells were incubated for 40 min at 37 °C. Nuclei were stained with 0.5 µM DAPI for 30 min at 37 °C in a humidified chamber protected from the light. The percentages of TUNEL-positive cells and the viral antigen-positive cells were recorded. Five representative photographs were taken from each coverslip using a Nikon C1 confocal laser scanning microscope. The images were acquired using NIS-Elements Advanced Research software (Nikon Instruments, Tokyo, Japan) and were analyzed with the ImageJ 1.44p Java 1.6.0_20 (32-bit) software.

### 2.19. DNA Fragmentation Assay

DNA fragmentation was assessed using the Apoptotic DNA Ladder Kit (Roche^®^ Cat. No. 11 835 246 001, Branchburg, NJ, USA). Rotavirus-infected cells (2.6 × 10^6^ cells/mL) were harvested at 12 and 24 h.p.i. and resuspended in PBS (200 µL) containing 0.5 mM PMSF and mixed with lysis buffer (200 µL) (6 M guanidine-HCl, 10 M urea, 10 mM Tris-HCl, pH 4.4, 20% Triton X-100). After incubation for 10 min at RT, isopropanol (100 µL) was added, followed by vortexing for 1 min. The filter and collection tubes provided in the kit were combined and the samples were applied into the upper reservoir and centrifuged at 6200× *g* for 1 min. The elute was discarded and the filter tube and used collector tube were combined before the addition of washing buffer (500 µL) (80% ethanol, 20 mM NaCl and 2 mM Tris-HCl, pH 7.5) into the upper reservoir. The combined tubes were centrifuged at 6200× *g* for 1 min; the fluid in the collection tube was discarded and the upper filter column was washed with 500 µL of washing buffer (20 mM NaCl and 2 mM Tris-HCl, pH 7.5, and 80% [*v*/*v*] ethanol). By combining the filter and collection tubes provided in the kit, the samples were applied into the upper reservoir and centrifuged at 6200× *g* for 1 min. The residual washing buffer was removed by centrifugation at 16,200× *g* for 10 s. After discarding the collection tube, the filter tube was inserted into a sterile Eppendorf tube and the DNA was eluted twice by adding 200 mL of pre-warmed (70 °C) elution buffer (10 mM Tris-HCl, pH 8.5) followed by centrifugation at 6200× *g* for 1 min. The eluted DNA was stored at −20 °C until its analysis. The purity and quantity of DNA was determined using a Nanodrop 2000c (Thermo Scientific, Rockford, IL, USA) and 2 mg of purified DNA in loading buffer (6X) (1% SDS, 0.05% bromophenol, 100 mM EDTA and 60% glycerol) were analyzed in a 1% agarose gel at 75 V (5 V/cm) constant voltage for 1.5 h. DNA was visualized with a SyBR^®^Safe DNA gel stain (Life Technologies, Cat. No. S33102, Rockford, IL, USA) using a UV transilluminator in a gel documentary system (Fotodyne UV Transilluminator 3-3000, Woonsocket, RI, USA). DNA from cells inoculated with UV-inactivated Wt1-5, non-infected cells, and H_2_O_2_-treated cells were used as a control.

### 2.20. Poly (ADP-Ribose) Polymerase-1 (PARP-1) Cleavage Assay

PARP-1 cleavage was assessed using rabbit-cleaved PARP-1 antibody (Santa Cruz Biotechnology Inc., SC-194C1439, Paso Robles, CA, USA). Cells infected with rotavirus Wt1-5 (MOI 0.1 to 6) in RPMI culture medium without FBS were harvested at 24 h.p.i., fixed in cold absolute ethanol for 30 min, collected by centrifugation at 400× *g* for 5 min and washed twice with PBS-T containing 1% BSA. Cells were plated onto coverslips and incubated with cleaved PARP-1 antibody (1 mL/mL) in blocking buffer (50 mM Tris-HCl, pH 8, 150 mM NaCl, 0.3% [*v*/*v*] Tween 20 and 5% skimmed milk) for 1 h at RT. After washing twice with PBS-T, the cells were incubated with secondary donkey anti-rabbit FITC-conjugated antibody (0.88 mg/mL, Santa Cruz SC-2024, Paso Robles, CA, USA) in PBS containing 1% BSA for 40 min at 37 °C in the dark, and then, washed twice with PBS before treatment with rabbit hyperimmune serum against rotavirus structural proteins. Cells were incubated for 1 h at 37 °C and washed twice with PBS before the addition of secondary goat anti-rabbit Alexa Fluor 568-conjugated antibody (0.88 mg/mL, Santa Cruz Biotechnology Inc., SC-2780, Paso Robles, CA, USA) in PBS containing 1% BSA and incubation for 40 min at 37 °C in the dark. DAPI (4′,6-diamidino-2-phenylindole) (0.5 µM) was added followed by incubation for 30 min at 37 °C in a humidified chamber protected from the light. Fixed and permeabilized cells were treated with recombinant DNase I, grade I (3 U/mL) in reaction buffer (50 mM Tris-HCl, pH 7.5, 10 mM MgCl_2_ and 1% BSA) for 10 min at RT to induce hydrolysis of phosphodiester linkages to generate positive control cells. Cells treated with H_2_O_2_ (1 mM) and non-infected cells were also used as a control. Positive cells for PARP-1 cleavage and for structural rotavirus antigens were recorded and expressed as a percentage of the total cell population observed. Representative photographs were taken using a Nikon C1 confocal laser scanning microscope. The images were obtained with the Nikon NIS-Elements Advanced Research software and analyzed with the ImageJ 1.44p Java 1.6.0_20 (32-bit) software (Nikon, Tokyo, Japan).

### 2.21. Cell Surface Membrane Proteins

Expression of cell surface membrane proteins Hsp90, Hsp70, Hsp60, Hsc70, PDI and integrin β3 was determined using flow cytometry. Reh cells at a logarithmic growth phase and PBMCs were fixed with 4% PFD for 30 min at RT and washed twice with PBS. Fixed cells were treated with 50 mM NH_4_Cl and 100 mM glycine in PBS for 30 min at RT before addition of goat polyclonal antibodies against Hsp90 (SC-1055), Hsp70 (SC-1060), Hsp60 (SC-1052), Hsp40 (SC-1801), Hsc70 (SC-1059), integrin β3 (SC-6627) or PDI (SC-17222) in PBS containing 1% BSA and incubation for 1 h at 37 °C. Following two washes in PBS, secondary donkey anti-goat FITC-conjugated antibody (0.88 mg/mL, Santa Cruz Biotechnology Inc., SC-2024, Paso Robles, CA, USA) in PBS containing 1% BSA were added before incubation for 1 h at RT in the dark. Fluorescence signals were detected using a BD FACSCanto II flow cytometer and analyzed using the FlowJo vX.0.7 software or FACSDiva software (Becton-Dickinson, Franklin lakes, NJ, USA). Isotype antibodies served as a negative control and median fluorescence intensity (MFI) values were determined for both positive and negative cell samples.

### 2.22. Colocalization Assay

Reh cells (3 × 10^5^) in 48-well plates (400 mL/well) were infected with rotavirus Wt1-5 at MOI 3 in RPMI culture medium without FBS and incubated for 1 h at 4 °C to bind the virus to cells without its internalization. The cells were then changed to 37 °C, harvested at 0, 5, 15, and 30 min post-infection and fixed with 4% PFD in PBS. The cells were plated onto coverslips, treated with goat antibodies (2 mg/mL) to Hsp90 (SC-1055), Hsp70 (SC-1060), Hsp60 (SC-1052), Hsp40 (SC-1801), Hsc70 (SC-1059), integrin β3 (SC-6627) or PDI (SC-17222) and rabbit antibodies to rotavirus structural proteins in PBS containing 1% BSA and were incubated for 1 h at 37 °C. After two PBS washes, the cells were incubated with secondary donkey anti-goat FITC-conjugated antibody (0.88 mg/mL, Santa Cruz Biotechnology Inc., SC-2024, Paso Robles, CA, USA) and secondary donkey anti-rabbit Alexa Fluor 568-conjugated IgG (0.88 mg/mL, Invitrogen A11004, Thermo Fisher, Rockford, IL, USA) in PBS containing 1% BSA and incubated for 40 min at 37 °C. Nuclei were stained with 0.5 µM DAPI and the coverslips were incubated for 30 min at RT protected from the light, washed twice with PBS and mounted on glass microscope slides in 70% glycerol in PBS and resin. Non-infected and infected cells without labels were used as a control, and isotype antibodies were used to adjust fluorescence intensity. Four representative photographs from each coverslip were taken using a Nikon C1 confocal laser scanning microscope controlled by Nikon’s EZ-C1 software Ver. Gold. 3.90 with Objective Plan. Apo. 100x/NA1.40/WD0.13 Oil PFS (Nikon Instruments, Tokio, Japan). A small pinhole diameter of 30 μm was used. Z-stack image acquisition was performed in a 16-bit format. Image analysis was conducted with the ImageJ 1.44p Java 1.6.0_20 (32-bit) software and colocalization was quantified by intensity correlation coefficient-based (ICCB) analysis [82]. The colocalization analysis of the rotavirus structural antigens and cell surface receptors was performed using the pixel intensity spatial correlation analysis and calculating Pearson’s correlation coefficients (PCC) and Mander’s overlap coefficient (MOC) [83]. The Z-stack images were obtained and a mean of 100 cells per variable assessed was analyzed using the region of interest (ROI) tool. The images of cells were obtained from three independent assays and one biological replicate. A perfect correlation was defined as +1, the absence of correlation as 0, and a perfect anti-correlation as −1. Mander’s overlapping coefficient was assumed as proportional to the amount of fluorescence of the colocalization pixels in each color channel. This coefficient varies from 0 to 1, where 0 corresponds to non-overlapping images and 1 to 100% colocalization of images [84].

### 2.23. Antibody Blocking Assay

Reh cells (2.0 × 10^5^ cells/300 µL) were seeded in each well of 48-well plates and incubated at 37 °C in a chamber with 5% CO_2_. The cells at a logarithmic growth phase were incubated with goat antibodies (4 or 0.4 mg/mL, Santa Cruz Biotechnology Inc. Paso Robles, CA, USA) against Hsp90 (SC-1055), Hsp70 (SC-1060), Hsp60 (SC-1052), Hsp40 (SC-1801), Hsc70 (SC-1059), integrin β3 (SC-6627) or PDI (SC-17222) or with a mixture of these seven antibodies (0.4 mg/mL, each antibody) in PBS containing 1% BSA for 1 h at 37 °C. The cells were washed two times with PBS to remove the unbound antibodies, and then, were immediately inoculated with rotavirus Wt1-5 (MOI of 2) as indicated above. The cells were collected at 24 h.p.i., fixed with 4% PFD in PBS for 30 min at RT, and then, washed twice with PBS. The fixed cells were treated with rabbit hyperimmune serum (1:2000 dilution) against rotavirus structural proteins in PBS containing 1% BSA for 1 h at 37 °C and washed twice with PBS. The cells were incubated with secondary donkey anti-rabbit FITC-conjugated antibody (0.88 mg/mL, Santa Cruz Biotechnology Inc., SC-2024, Paso Robles, CA, USA) in PBS containing 1% BSA for 30 min at 37 °C in the dark. Fluorescence signals were detected using a BD FACSCanto II flow cytometer. The data were analyzed using the FlowJo vX.0.7 software or FACSDiva software (Becton-Dickinson, Franklin lakes, NJ, USA). Isotype antibodies were used as a control for excluding non-specific fluorescence signals. Median fluorescence intensity (MFI) values were determined for both positive and negative cell samples.

## 3. Results

### 3.1. Infection of Reh Cells by Rotavirus Isolate Wt1-5

**Figure 1:**
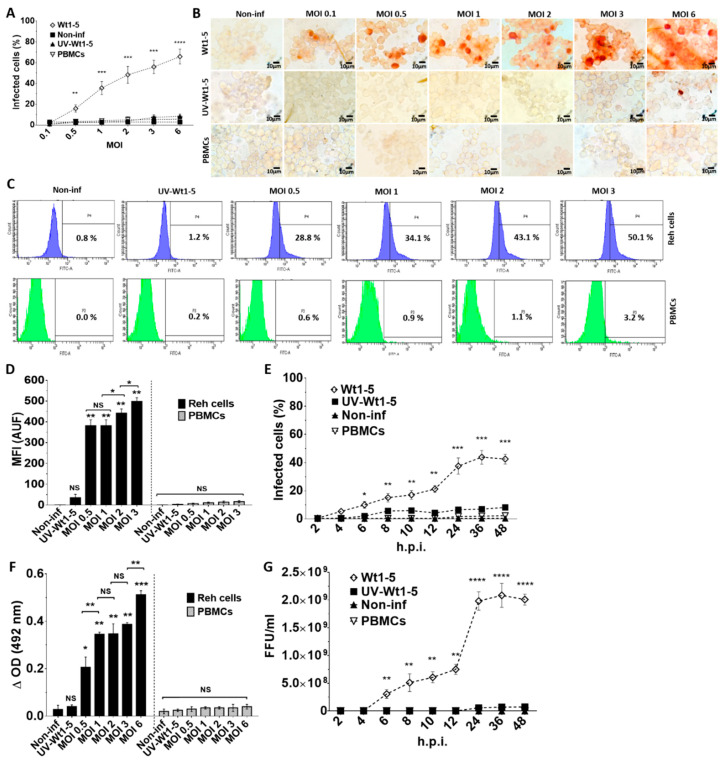
Infection of Reh cells and PBMCs by rotavirus isolate Wt1-5. Reh cells at a logarithmic growth phase were infected with trypsin-activated Wt1-5 (MOI 0.1 to 6) (or left uninfected) in RPMI culture medium without FBS. PBMCs were used as a control. UV-inactivated Wt1-5-infected cells were used as a control. (**A**) Samples of Reh cells and PBMCs were collected at 24 h.p.i. and subjected to immunochemistry assay using antibodies to rotavirus structural proteins. Percentages of infected cells at different MOIs are shown. (**B**) Representative photographs of immunochemistry assays at 24 h.p.i. at different MOIs are shown. (**C**) Flow cytometric analysis of Wt1-5-infected Reh cells and PBMCs for rotavirus antigen at different MOIs is depicted. (**D**) Quantification of fluorescence intensity in arbitrary units of fluorescence (AUF) for rotavirus antigens is shown for infected Reh cells and PBMCs (**E**) Immunocytochemistry analysis for rotavirus antigens of infected Reh cells and PBMCs at MOI 2 through the post-infection time (2 to 48 h) is shown. (**F**) ELISA results showing the presence of rotavirus antigens in the 700 g supernatant of infected Reh cells and PBMCs at 24 h.p.i. are shown. (**G**) Infection of Reh cells and PBMCs with trypsin-activated 700 g supernatants obtained from lysates of previously infected cells taken at the post-infection times indicated in (**E**). Virus titers (FFU/mL) were determined by immunochemistry at 12 h.p.i. Data are presented as mean ± SD of three independent experiments performed in duplicate. Statistical significance is indicated by *p*-values (**** *p* ≤ 0.001, *** *p* ≤ 0.01, ** *p* ≤ 0.05, and * *p* ≤ 0.1).

Rotaviruses appear to enter cells by a complex mechanism that involves sequential or alternative interactions with cell surface molecules. We wanted to know whether tumor-adapted rotavirus Wt1-5 had developed a special tropism for Reh cells. To test for this tropism, Reh cells growing at a logarithmic phase were inoculated with the tumor cell-adapted rotavirus isolate Wt1-5 at MOIs of 0.1 to 6 in culture medium without FBS. Both Reh cells and control PBMCs infected with Wt1-5 were subjected to immunochemistry analysis at 24 h.p.i. for the presence of rotavirus structural proteins. The percentage of rotavirus antigen-positive cells was found to be increased with the increasing MOI in Reh cells, while PBMCs lacked viral antigens even at the highest MOIs used (Figure 1A). The rotavirus-positive cells were evident in comparison with the cells that had been inoculated with UV-inactivated Wt1-5 or Wt1-5 inoculated-PBMCs used as a control. The results are observed in the immunocytochemistry images (Figure 1B). The flow cytometry analysis showed that, in comparison with the UV-inactivated virus-infected Reh cells and Wt1-5 infected PBMCs, Reh cell infection with trypsin-activated Wt1-5 resulted in increasing proportions of rotavirus antigen-positive cells according to the increasing MOIs used (Figure 1C). The increasing median fluorescence intensity (MFI) for the viral antigen-positive cells according to the increasing MOIs used was also evident after the flow cytometry analysis, while no increase in MFI was found for Wt1-5-infected PBMCs (Figure 1D). The time-course of viral antigen accumulation in infected cells through 2 to 48 h.p.i. also showed that rotavirus isolate Wt1-5 was certainly multiplied in Reh cells but was not in Wt1-5 infected PBMCs (Figure 1E). Rotavirus structural antigens were detected by ELISA in the 700 g supernatant fraction from infected cells harvested at 24 h.p.i. Viral antigens in the supernatant showed an enhanced accumulation that was associated with the increasing MOIs used in the infection assay (Figure 1F). No significant statistical differences were observed for the viral antigens detected in the supernatant when Wt1-5-infected PBMCs and non-infected Reh cells were compared. The infection assay using inocula consisting of trypsin-activated extracts from aliquots taken at the post-infection time points described in Figure 1E showed that these inocula were able to produce new infectious viral particles after 12 h post-infection when inoculated in new Reh cells but were not in Wt1-5-infected PBMCs (Figure 1G). All analyses and SD values were based on three independent experiments performed in duplicate. These results strongly suggest that Wt1-5 can successfully and selectively infect Reh cells but cannot PBMCs, indicating a significant tropism Wt1-5 for Reh cells.

### 3.2. Effects of Wt1-5 on Cell Viability and Cell Membrane Permeability

**Figure 2:**
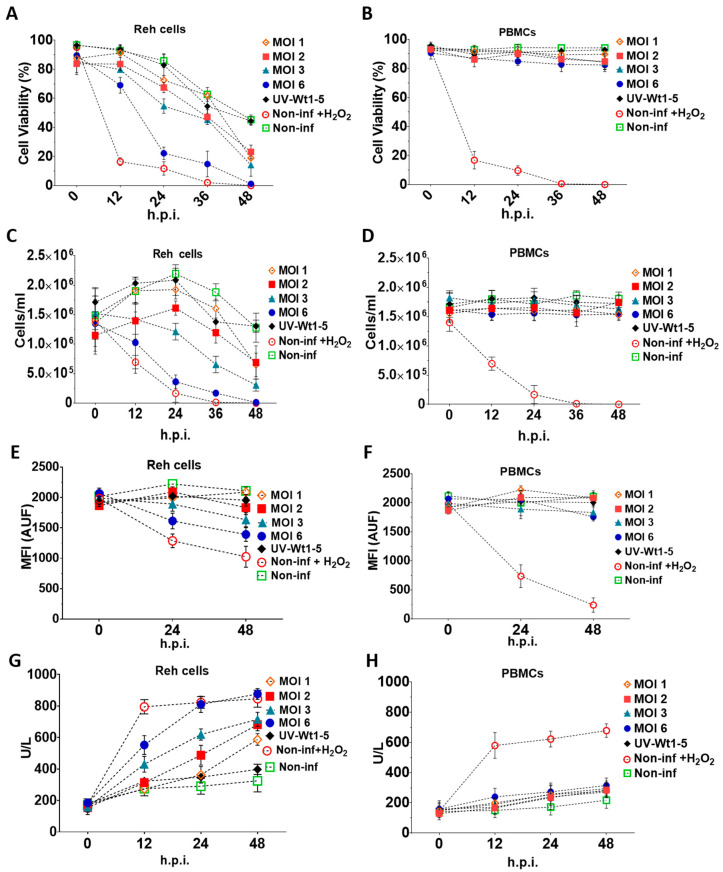
Effects of Wt1-5 infection of Reh cells and PBMCs on cell viability and cell membrane permeability. Reh cells at a logarithmic growth phase and PBMCs were inoculated with trypsin-activated Wt1-5 (MOI 1 to 6) in RPMI culture medium without FBS. Non-infected cells, UV-inactivated virus-infected cells at MOI 2, and non-infected and H_2_O_2_-treated cells were used as a control. Infected Reh cells (**A**) and PBMCs (**B**) were harvested at the indicated post-infection times (h.p.i.) and subjected to the trypan exclusion test. Reh Cells (**C**) and PBMCs (**D**) were collected at the post-infection times indicated and the viable cells counted in a Neubauer chamber and expressed as cells/mL. Cell proliferation analysis of Reh cells (**E**) and PBMCs (**F**) infected or not infected with Wt1-5 (MOIs 1 to 6), using CellTracker™ Blue CMAC Dye. Quantification is expressed in terms of arbitrary fluorescence units (AUF) through the post-infection period indicated. Cell membrane permeability of Reh Cells (**G**) and PBMCs (**H**) was determined at the indicated post-infection times by measuring lactate dehydrogenase (LDH) activity in the 700 g supernatant of infected, non-infected and UV-inactivated virus-infected cells cultured in RPMI culture medium without FBS. Data are shown as mean SD of three independent experiments performed in duplicate.

To assess the effects of Wt1-5 on cell viability and cell membrane permeability, Reh cells at a logarithmic growth phase and PBMCs were infected with trypsin-activated Wt1-5 (MOI 1 to 6) in the absence of FBS. The trypan blue exclusion test showed that the Reh cell viability was continuously decreased through the post-infection time points studied (0, 12, 24, 36 and 48 h.p.i.) for all the tested conditions (Figure 2A). In contrast, PBMCs maintained their cell viability within a range between 88–95%, which was similar to that of non-infected Reh cells regardless of the MOI used (Figure 2B). However, the cell viability was drastically reduced from the 12 h.p.i. in the case of the H_2_O_2_-treated control cells, while the effect of Wt1-5 (MOI of 6) infection on cell viability slightly approached that of H_2_O_2_ treatment only after 24 h.p.i. In both cases, cell viability was nearly reduced from 20% to 0% between 24 and 48 h.p.i. (Figure 2A). The lowest decrease in cell viability was observed in the case of non-infected cells, where the percentage of viable cells ranged from about 90% to 50% through the same post-infection period (Figure 2A). The cell viability observed in cells inoculated with UV-inactivated Wt1-5 (MOI of 2) ranged from 80% at 24 h.p.i. to 50% at 48 h.p.i., while the cell viability for cells infected with Wt1-5 (MOI of 1, 2 and 3) ranged from about 70% to 30% through the same post-infection period. These cell viability values showed significant differences between the activated and inactivated virus (Figure 2A). To quantify the number of cells remaining in the culture for Reh cells (Figure 2C) and PBMCs (Figure 2D) through the post-infection time examined, a Neubauer chamber was used. In the case of infected Reh cells, the results indicated that significantly less morphologically identifiable cells remained between 12 and 48 h.p.i. in the cases of H_2_O_2_-treated cells and Wt1-5-infected cells at MOI 6 when compared with non-infected cells or cells infected with UV-inactivated Wt1-5 in terms of cells/mL (Figure 2C). Intermediate cell concentrations were observed for cells infected with Wt1-5 at MOIs of 1, 2 and 3 (Figure 2C). Except for H_2_O_2_-treated cells and Wt1-5-infected cells at MOI of 3 and 6, the cells infected with UV-inactivated Wt1-5, cells infected with Wt1-5 (MOIs of 1 and 2) and non-infected cells showed an increase in cell concentration between 0 and 24 h.p.i., probably due to cell proliferation induced by residual FBS (Figure 2C). In the case of PBMCs infected or not with Wt1-5, the number of cells remained almost the same throughout the culture period examined, regardless of MOIs used. However, PBMCs treated with H_2_O_2_ reduced their cell viability by more than 60% at 12 h posttreatment and 90% at 24 h posttreatment (Figure 2D). These results reveal that Wt1-5 infection decreases significantly both cell viability and cell number of Reh cells after 12 h.p.i. and that this effect is more evident at higher MOI values and longer post-infection times. These effects were absent in the case of the control PBMCs.

Reh cells (Figure 2E) and PBMCs (Figure 2F) labeled with fluorescent dye CellTracker™ Blue CMAC Dye (Life Technologies), a fluorescent thiol-reactive cell-permeant probe, were inoculated with Wt1-5 (MOIs 1 to 6) in RPMI culture medium without FBS. The analysis of the intracellularly labeled cells revealed that the proliferation of infected Reh cells at MOI 6 was decreased by 30% and 37% at 24 and 48 h.p.i., respectively, in terms of AUF. Fluorescence remained constant for control cells consisting of cells infected with UV-inactivated virus or non-infected cells. In contrast, relative fluorescence was decreased by 40% and 50% in H_2_O_2_-treated Reh cells at 24 and 48 h.p.i., respectively. Cell proliferation of infected cells at MOI 3 was decreased by about 20% at 48 h.p.i., whereas infection of cells using an MOI of 1 or 2 did not affect significantly cell proliferation as compared with control cells (Figure 2E). The inoculation of PBMCs with Wt1-5 did not produce a decrease in the fluorescence intensity of CellTracker™ Blue CMAC Dye (Figure 2F). Cell viability was further assessed by quantifying LDH activity in Reh cells (Figure 2G) and PBMCs (Figure 2H). The analysis indicated that the Reh cell viability profiles were very similar to that found using the trypan blue exclusion test at the indicated post-infection times, in which the higher effect on cell viability was caused by H_2_O_2_ treatment or Wt1-5 infection at the highest MOI used, in comparison to the cell viability in the case of non-infected cells (Figure 2G). In the case of PBMCs, no statistically significant differences in LDH activity were found when cells infected with Wt1-5 were compared with those that had not been infected. The LDH activity present in the 700 g supernatant fraction suggests that cell membrane permeability is significantly and selectively affected by Wt1-5 infection of Reh cells in an MOI-dependent manner, while the cell membrane permeability of PBMCs was not affected.

### 3.3. Effect of Wt1-5 Infection on Redox Activity, Mitochondrial Membrane Potential (∆Ψm) and Cell Membrane Permeability

**Figure 3:**
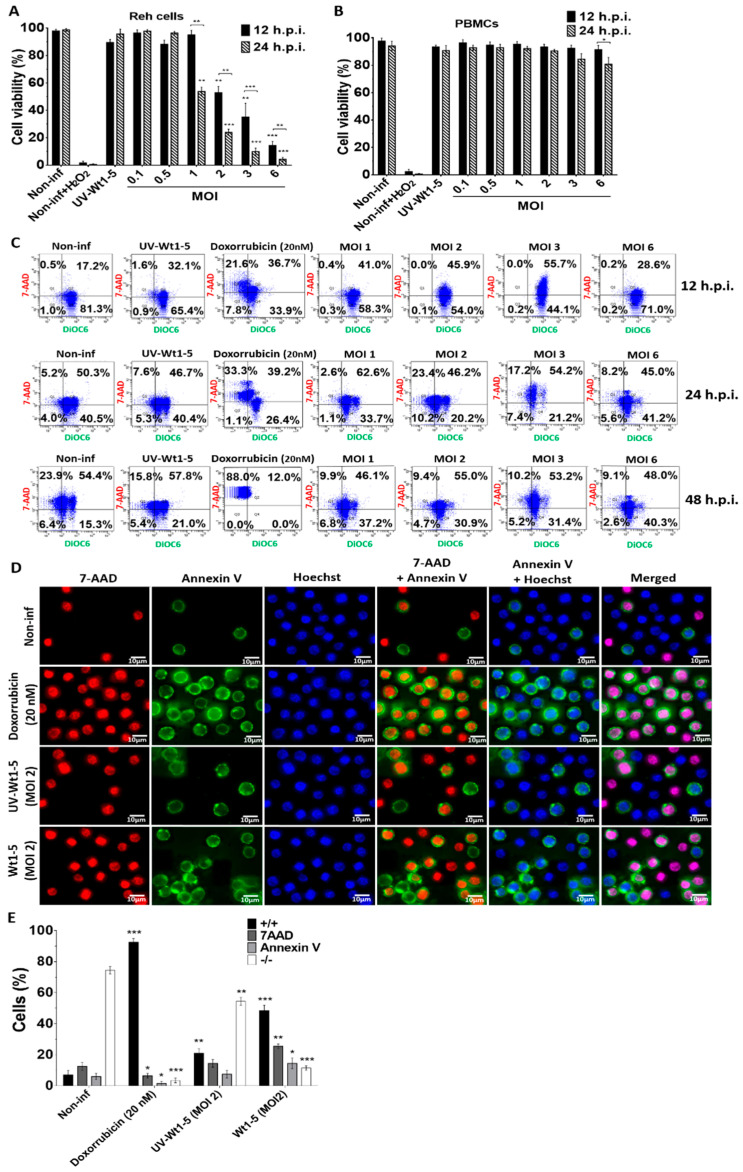
Effect of Wt1-5 infection on redox activity, mitochondrial membrane potential (∆Ψm) and cell membrane permeability. Reh cells at a logarithmic growth phase were infected or not infected with trypsin-activated Wt1-5 (MOI 1 to 6) in RPMI culture medium without FBS. UV-inactivated Wt1-5-infected cells and non-infected cells treated with H_2_O_2_ (1 mM) or doxorubicin (20 nm) were used as a control. Cell viability was determined by measuring the cell oxidoreductive activity using the resazurin reduction test at 12 and 24 h.p.i. (**A**) Infected Reh Cells and (**B**) PBMCs are shown. (**C**) Flow cytometry analysis of mitochondrial membrane potential (∆Ψm) using DiOC6 (3) (green), and cell membrane permeability using 7-AAD (red). Cells were analyzed at 12, 24 and 48 h.p.i. Dot plots represent the combination of both fluorescent signals. Lower right quadrant: viable cells (non-apoptotic and non-necrotic); upper right quadrant: necrotic cells; lower left quadrant: early apoptotic cells; upper left quadrant: late apoptotic cells. (**D**) Externalized phosphatidylserine was visualized with annexin V (FITC (green), cell membrane permeability was determined with 7-AAD (red) and nuclei were stained with Hoechst 33342 (in blue). Representative photographs are shown at 24 h.p.i. (**E**) Quantitative analysis of images shown in c. (+/+): cells positive for both annexin V and 7-AAD; (−/−): cells negative for both stainings. Image analysis was conducted using ImageJ 1.44p Java 1.6.0_20 (32-bit) software. Data are shown as mean ± SD of three independent experiments performed in duplicate. Statistical significance is indicated by *p*-values (*** *p* ≤ 0.01, ** *p* ≤ 0.05, and * *p* ≤ 0.1).

Reh cell (Figure 3A) and PBMCs (Figure 3B) infected with trypsin-activated Wt1-5 (MOIs 1 to 6) in the absence of FBS as indicated above were analyzed for its effect on redox activity, mitochondrial membrane potential (∆Ψm) and cell membrane permeability. After the resazurin treatment of infected Reh cells at the indicated post-infection times, the fluorescence analysis revealed that both at 12 and 24 h.p.i., the mitochondrial function and cell viability were dramatically decreased by Wt1-5 infection, which was more evident at higher MOI values and longer post-infection times (24 h.p.i.) (Figure 3A). The analysis of cell viability used non-infected cells, H_2_O_2_-treated cells and UV-inactivated virus-infected cells as controls. The same analysis performed on Wt1-5-infected PBMCs showed no reduction in mitochondrial oxidoreductive activity, except at 24 h.p.i., when cell viability was decreased by 18% in PBMCs at an MOI value of 6, in comparison to non-infected PBMCs (Figure 3B). The mitochondrial membrane potential (∆Ψm) and the Reh cell membrane permeability were assessed using DiOC6(3) and 7-AAD, respectively, at 12 and 24 h.p.i. The flow cytometry dot plots represent the combination of both stainings, in which viable non-apoptotic/non-necrotic cells are in the lower right quadrant (Figure 3C). The upper right quadrant represents necrotic cells, while the lower left quadrant represents early apoptotic cells and the upper left one represents late apoptotic cells (Figure 3C). These results indicated that at 12 h.p.i., 81.3% of non-infected control cells kept their ∆Ψm as suggested by their positive signals for DiOC6(3), while the proportion of 7-AAD-positive cells was very low. However, the percentage of 7-AAD-positive cells was significantly increased at 24 and 48 h.p.i., suggesting that the viability of Reh cells was dependent on FBS, as indicated in the results previously described in Figure 2A,B. Similar results were found for cells inoculated with UV-inactivated virus, while doxorubicin-treated cells showed percentages of 7-AAD-positive cells at 12 and 24 h.p.i that were similar to those shown by non-infected cells. Most of the doxorubicin-treated cells (88%) were 7-AAD-positive at 48 h.p.i. (Figure 3C). On the other hand, the Wt1-5-infected cells showed an increased percentage of cells being positive to 7-AAD and DiOC6(3), which was positively correlated with increasing post-infection time and MOI (Figure 3B). However, this trend was not very systematic as some longer post-infection times and higher MOIs did not necessarily produce higher percentages of cells being simultaneously positive to 7-AAD and DiOC6(3) (Figure 3C). Unexpectedly, an increase in the percentage of viable cells was recorded at 48 h.p.i. This could be explained by a decrease in the number of infected cells undergoing lysis due to viral infection (e.g., infected cells die and non-infected cells remain or proliferate by residual FBS stimulus).

Early apoptotic signals induced by Wt1-5 infection on Reh cells were assessed by detecting phosphatidylserine exposure in the outer cell membrane. Cell membrane permeability changes were assessed using 7-AAD staining, while nuclei were stained with Hoechst 33342. Representative images of these stainings analyzed at 24 h.p.i. are shown in Figure 3D. Viable cells were only positive for Hoechst 33342 staining (blue), while early apoptotic cells were positive for both annexin V (green) and Hoechst 33342 staining (blue). On the other hand, cells exposing phosphatidylserine in their outer cell membrane, but being at late apoptosis, were positive for annexin V (green), 7-AAD (red) and Hoechst 33342 (blue) staining. Cells that had lost their membrane integrity and had no phosphatidylserine exposure were only positive for 7-AAD (red) and Hoechst 33342 (blue) staining (Figure 3D). The percentages of cells being positive for the different staining described in Figure 3D are shown in Figure 3E. Doxorubicin-treated cells, non-infected cells, and cells infected with UV-inactivated Wt1-5 were used as controls. Most of the rotavirus-infected cells at MOI 2 were doubly positive for fluorescent signals from 7-AAD and annexin V (Figure 3E), while the non-infected control cells were negative for both stainings. The cells inoculated with UV-inactivated Wt1-5 were slightly positive for both fluorescent labels, whereas the doxorubicin-treated cells were predominantly positive for both fluorescent signals. However, the fluorescent signals did not allow for determining of the percentage of rotavirus-infected cells.

### 3.4. Induction of Cell Death Markers by Wt1-5 Infection

**Figure 4:**
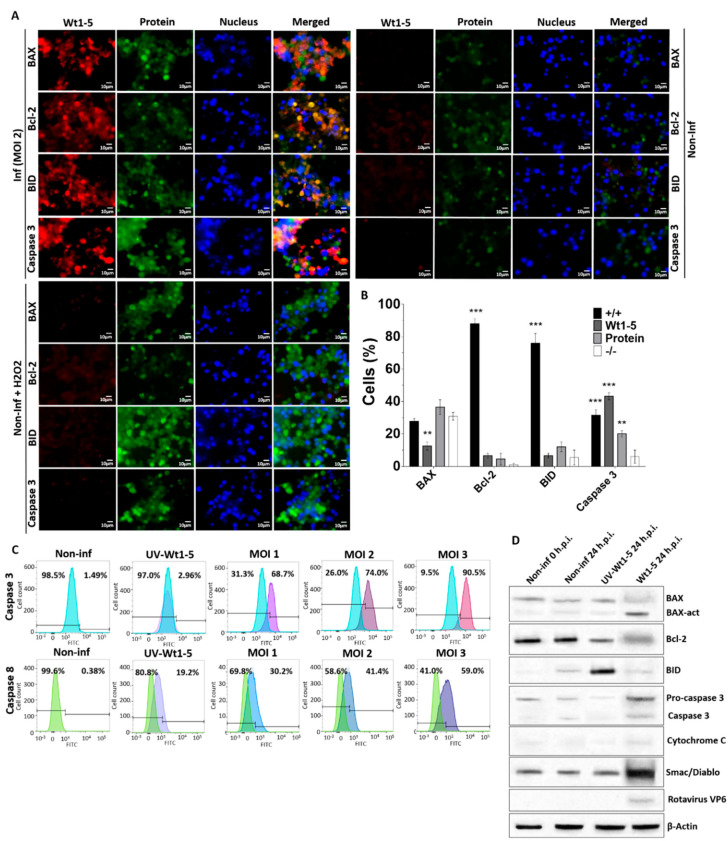
Cell death markers induced by Wt1-5 infection of Reh cells. Reh cells at a logarithmic growth phase were infected or not infected with trypsin-activated Wt1-5 (MOI 2) in RPMI culture medium without FBS. Non-infected cells treated with H_2_O_2_ (1 mM) were used as a control. (**A**) Representative photographs of expression for cellular proteins BAX, Bcl-2, BID and Caspase 3 are shown at 24 h.p.i. Cellular proteins were stained with Alexa Fluor 488 (green) and viral structural antigens with Alexa Fluor 568 (red). Nuclei were stained with DAPI (blue). (**B**) Quantitative analysis of images shown in (a) is expressed in terms of percentages of cells being positive for cellular proteins or Wt1-5 structural proteins. (+/+): cells positive to both viral proteins and cellular proteins; (−/−): cells negative for both viral proteins and cellular proteins. (**C**) Flow cytometry analysis at 24 h.p.i. of Wt1-5-infected cells at MOI 1–3 for activation of caspases 3 and 8. Non-infected cells and UV-inactivated Wt1-5-infected cells at MOI 2 were used as a control. Histograms for non-infected and infected cells were overlapped. Percentages of positive cells are on the right and negative ones on the left. Results are from two different assays. (**D**) Lysates from Wt1-5-infected cells at MOI 2 were analyzed by SDS-PAGE/Western blotting at 24 h.p.i. using antibodies to BAX, activated-BAX, Bcl-2, BID, pro-caspase 3, caspase 3, cytochrome C, Smac/Diablo, rotavirus VP6 and β-actin. Lysates from non-infected cells at 0 and 24 h.p.i. were used as a control. Data are shown as mean ± SD of three independent experiments performed in duplicate. Statistical significance is indicated by *p*-values (*** *p* ≤ 0.01, ** *p* ≤ 0.05, and * *p* ≤ 0.1).

To assess the expression of cell death markers, an analysis of BAX, Bcl-2, BID and caspase 3 expression was conducted at 24 h.p.i. using immunofluorescence microscopy of Wt1-5-infected cells at MOI 2. Rotavirus structural proteins were detected with Alexa Fluor 568-labeled antibodies (red), while BAX, Bcl-2 and caspase 3 were detected with Alexa Fluor 488-labeled antibodies (green) and nuclei were visualized with DAPI (blue) (Figure 4A). Fluorescent signals for cell death markers from non-infected control cells were extremely weak, whereas such signals were greatly enhanced for BAX, BID and caspase 3 from Wt1-5-infected cells with no changes in Bcl-2 (Figure 4A). Non-infected and H_2_O_2_-treated cells also showed enhanced fluorescent signals for BAX, BID and caspase 3, except for Bcl-2 (Figure 4A). The association between virus infection and expression of cell death markers were evident following the calculation of percentages of cells being positive or negative for the cell death markers studied and the viral structural proteins (Figure 4B). This analysis showed that cells were simultaneously positive for viral structural proteins and BID or Bcl-2 were predominantly observed in the cell populations examined, whereas very low percentages of cells being positive for either cellular protein were detected. The simultaneous presence of either BAX or caspase 3 and viral structural proteins was detected in a significantly lower percentage of cells than that found for either cellular protein (BID or Bcl-2) alone. The percentages of cells showing positive signals only for either BAX or caspase 3 were similar to those found for cells being simultaneously positive for viral structural proteins and either BAX or caspase 3 (Figure 4A).

To further analyze the effect of Wt1-5 infection on cell death markers, infected Reh cells at MOI 2 were tested for caspases 3 and 8 and their flow cytometry histograms for non-infected and infected cells were overlapped. The analysis of positive and negative cells indicated that the percentages of expression of both caspase 3 and 8 were enhanced by virus infection, but the increase in caspase 3 was more pronounced than that of caspase 8 (Figure 4C). The increased expression of these caspases was positively related with increasing MOIs as compared with non-infected control cells and UV-inactivated virus-infected cells (Figure 4C). Caspase 3 expression was further analyzed using SDS-PAGE/Western blotting of lysates from Wt1-5-infected cells (MOI 2) at 24 h.p.i. This analysis used antibodies against cellular proteins BAX, activated BAX, Bcl-2, BID, pro-caspase 3, activated caspase 3, cytochrome C, Smac/Diablo, rotavirus VP6 and β-actin (Figure 4D). Infected cells showed a relatively increased expression of activated BAX, activated caspase 3 and Smac/Diablo, while Bcl-2 showed decreased expression in comparison to that of non-infected control cells. Cells inoculated with UV-inactivated Wt1-5 showed an increased expression of BID (Figure 4D). Overall, these results suggest that Wt1-5 infection of Reh cells can induce direct and indirect cell death mechanisms.

### 3.5. Induction of DNA Fragmentation by Wt1-5 Infection

**Figure 5:**
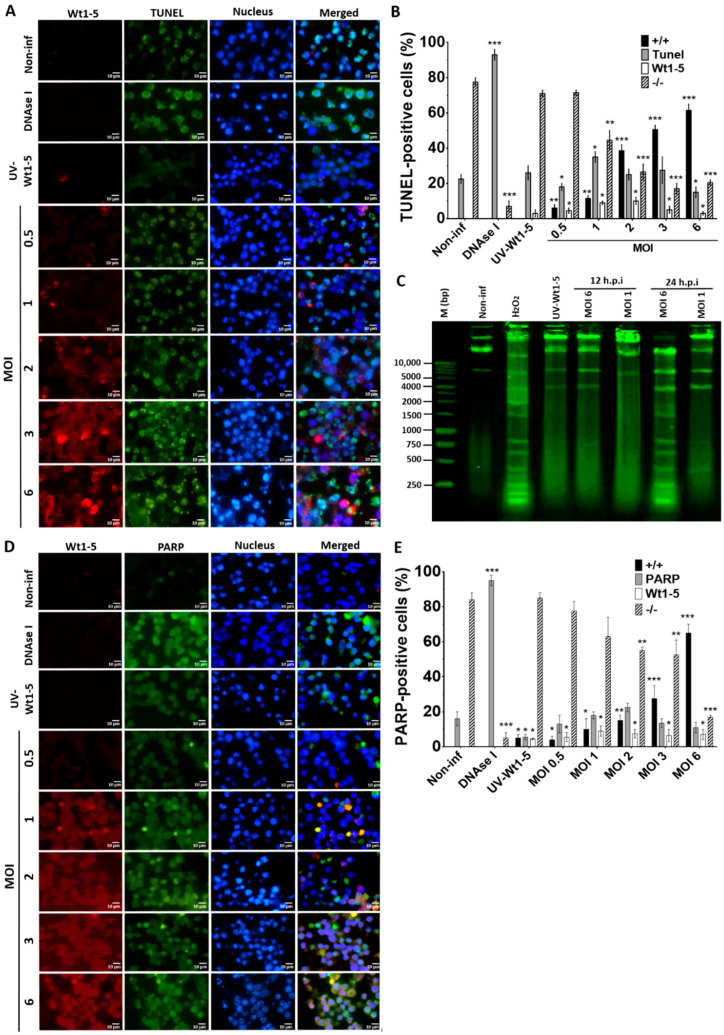
Nuclear fragmentation induced by Wt1-5 infection of Reh cells. Reh cells at a logarithmic growth phase were infected or not infected with trypsin-activated Wt1-5 (MOI 0.5 to 6) in RPMI culture medium without FBS. UV-inactivated Wt1-5-infected cells and DNAse I-treated cells after permeabilization were used as a control. (**A**) Representative photographs of immunofluorescence analysis for TUNEL assays are shown. Cells were labeled with Alexa Fluor 488 (green) for TUNEL, Alexa Fluor 568 (red) for Wt1-5 structural proteins and DAPI (blue) for nuclei. (**B**) Quantitative analysis of images shown in (A) is presented as percentages of cells being positive to TUNEL at the indicated MOIs at 24 h.p.i. (+/+): positive TUNEL cells that are also positive for Wt1-5 structural proteins; (−/−): cells negative to both TUNEL and Wt1-5 structural proteins. (**C**) DNA fragmentation pattern from Wt1-5-infected cells at MOI 1 and 6 is shown in an agarose gel (1%) after staining with SyBR^®^ Safe DNA gel stain at 12 and 24 h.p.i. (**D**) Representative photographs of immunofluorescence assays for PARP-1 cleavage at 24 h.p.i. are shown. Cleaved PARP-1 was labeled with FITC (green), Wt1-5 structural proteins with Alexa Fluor 568 (red) and nuclei with DAPI (blue). (**E**) Quantitative analysis of images presented in (d) is shown as percentages of cleaved PARP-1-positive cells. (+/+): positive cells to both cleaved PARP-1 and Wt1-5 structural proteins; (−/−): cells negative to both cleaved PARP-1 and Wt1-5 structural proteins. Data are shown as mean ± SD of three independent experiments performed in duplicate. Statistical significance is indicated by *p*-values (*** *p* ≤ 0.01, ** *p* ≤ 0.05, and * *p* ≤ 0.1).

To examine how Wt1-5 infection could induce cell death, Reh cells at a logarithmic growth phase were infected with the trypsin-activated virus at several MOIs (0.5 to 6) and cultured in the absence of FBS. The fixed and permeabilized cells harvested at 24 h.p.i. were stained with FITC-labelled dUTP (green) for DNA, Alexa Fluor 568 (red) for rotavirus structural proteins, and DAPI (blue) for nuclei. We found that in comparison to UV-inactivated virus-infected cells, non-infected cells or non-infected DNAse-treated cells, the percentage and intensity of TUNEL-positive cells following Wt1-5 infection were significantly increased (Figure 5A). The quantification of cells being simultaneously positive for TUNEL (green) and rotavirus antigens (red) indicated that their percentage was increased proportionally to the MOI used, while concomitantly, the percentage of cells being simultaneously negative for both TUNEL and rotavirus decreased with increasing MOI (Figure 5B). Since apoptosis involves cleavage of DNA into mono- and oligonucleosomal DNA fragments, DNA purified from Wt1-5 infected cells at 12 and 24 h.p.i. was analyzed by agarose gel electrophoresis. The results indicated that Wt1-5 infection resulted in DNA fragmentation that was compatible with an apoptotic DNA fragmentation pattern in comparison to agarose electrophoretic patterns for DNA from non-infected cells and H_2_O_2_-treated cells used as a control (Figure 5C). We detected the levels of the 89 kDa fragment of PARP-1 using a secondary FITC-conjugated antibody (green) in Wt1-5-infected Reh cells at 24 h.p.i. The rotavirus structural antigens were labeled with Alexa Fluor 568 (red). The results indicated that PARP-1 increased its cleavage with increasing MOI when compared to non-infected control cells (Figure 5D). However, PARP-1 cleavage in infected cells was lower than that observed in H_2_O_2_-treated control cells. The percentage of cells being simultaneously positive to PARP-1 cleavage and rotavirus antigens increased with MOI values (Figure 5E). The quantitative data and their SD were from three independent results performed in duplicate. These results appear to indicate that Wt1-5 infection can induce DNA fragmentation, a key feature of apoptotic cell death.

### 3.6. Cell Surface Proteins and Wt1-5 Infection

**Figure 6:**
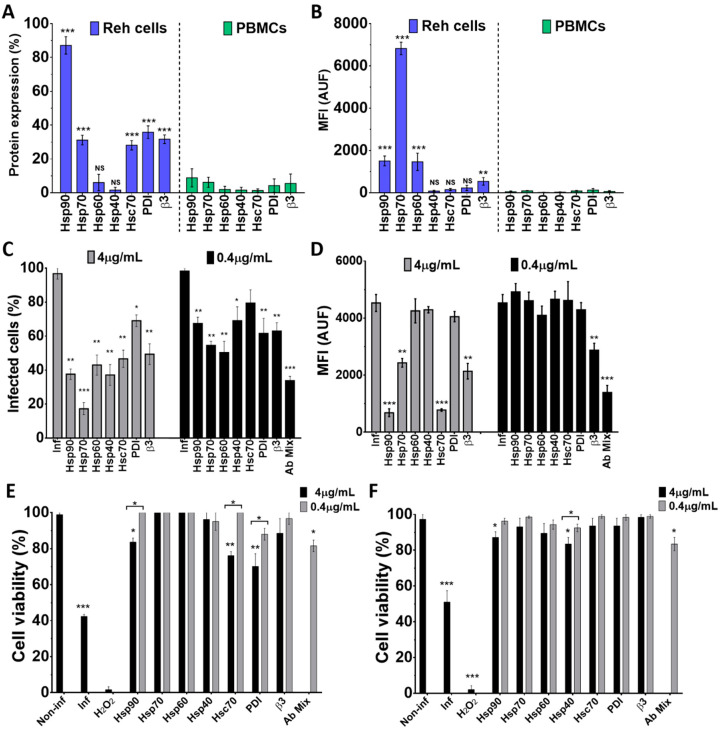
Inhibition of Wt1-5 infection after pre-treatment of Reh cells with antibodies to HSPs, Hsc70, PDI and integrin β3. Reh cells and PBMCs were treated with antibodies to Hsp90, Hsp70, Hsp60, Hsp40, Hsc70, PDI and integrin β3 and labeled with secondary antibodies labeled with FITC, and then, subjected to flow cytometric analysis. (**A**) Flow cytometry analysis for each cellular protein of Reh cells and PBMCs is shown. (**B**) Median fluorescence intensity (MFI) for each cellular protein of Reh cells and PBMCs is shown. Isotype antibodies were used to adjust quadrants. Results are from three different assays. (**C**) Reh cells at a logarithmic growth phase were pre-treated with antibodies (4 or 0.4 mg/mL) to cellular proteins Hsp90, Hsp70, Hsp60, Hsp40, Hsc70, PDI and integrin β3 for 1 h at 37 °C. After removal of antibodies, cells were infected with trypsin-activated Wt1-5 at MOI 2 and harvested at 24 h.p.i. before PFD fixation and permeabilization. Viral structural antigens were analyzed by flow cytometry for each antibody treatment using control isotype antibodies to adjust quadrants. Percentages of Wt1-5-infected cells are shown. (**D**) Median fluorescence intensity (MFI) quantification in terms of arbitrary units of fluorescence (AUF) is shown for viral structural proteins for each antibody treatment described in (**C**). (**E**) Cell viability was assessed with the resazurin reduction test for cells that had previously been treated with antibodies to each cellular protein (Hsp90, 7Hsp0, Hsp60, Hsp40, Hsc70, PDI and integrin β3). Wt1-5 infected cells and non-infected and non-antibody-treated cells were used as a control at 24 h.p.i. (**F**) Cell viability of an aliquot of cells assayed in (**E**) but determined with the trypan blue exclusion test is shown. Data are shown as mean ± SD of three independent experiments performed in duplicate. Statistical significance is indicated by *p*-values (*** *p* ≤ 0.01, ** *p* ≤ 0.05, and * *p* ≤ 0.1).

To assess the role of some cellular proteins in the Wt1-5 infection of Reh cells and PBMCs, the expression profile of cellular proteins HSPs (90, 70, 60 and 40), Hsc70, PDI and integrin β3 was examined using antibodies directed against these proteins. Using flow cytometry density dot plots, it was found that all the tested proteins showed detectable levels, but the expression of HSPs showed great variation (Figure 6A). In Reh cells, the percentages of expression levels were distributed in the following order: Hsp90 (92.3%), PDI (39.6%), Hsc70 (30.9%), integrin β3 (29.1%), Hsp70 (28.3%), Hsp60 (10.9%) and Hsp40 (3.2%). In the case of PBMCs, the mean percentages of protein expression were: Hsp90 (9.5%), Hsp70 (7.2%), integrin β3 (5.1%), PDI (4.2%), Hsp60 (1.8%), Hsc70 (1.2%), and Hsp40 (0.8%) (Figure 6A). In Reh cells, the highest MFI value was observed for Hsp70, followed by Hsp90 and Hsp60, while the lowest values were observed for Hsp40, Hsc70, integrin β3 and PDI. In contrast, all the cellular proteins assessed for PBMCs showed very low MFI values (Figure 6B). Pre-treatment of Reh cells with polyclonal antibodies to HSPs (90, 70, 60 and 40), Hsc70, PDI and integrin β3 before Wt1-5 inoculation at MOI 2 indicated that viral structural antigens were reduced at 24 h.p.i. when antibodies to all of the tested proteins were added separately at 4 μg/mL (Figure 6C). The highest negative effect on the accumulation of rotavirus structural proteins was caused by antibodies directed against Hsp70, which reduced them by 82% relative to normalized non-antibody-treated cells. Antibodies to Hsp90 or PDI reduced viral structural protein accumulation by 65% and 26%, respectively (Figure 6C). Pre-treatment of cells with diluted antibodies (0.4 μg/mL) produced similar inhibitory effects to those observed with non-diluted antibodies (4 μg/mL) (Figure 6C). Unexpectedly, the antibody mixture (0.4 μg/mL each) did not produce additive inhibitory effects on viral structural protein accumulation (Figure 6C). The inhibitory effects of cell antibody pre-treatment on the accumulation of rotavirus structural proteins were also estimated in terms of MFI arbitrary units (Figure 6D). This estimation showed that significant inhibition of viral structural protein accumulation was only caused by pre-treatment with antibodies to Hsp90, Hsc-70, Hsp70, and integrin β3 when used at 4 μg/mL. Diluted antibodies (0.4 μg/mL) did not reduce viral production, except those directed to integrin β3, and also, the mixture of all the antibodies tested (Figure 6D).

To test for the effect of antibodies against cellular proteins on cell viability, cells were separately incubated with antibodies against Hsp90, Hsp70, Hsp60, Hsp40, Hsc70, PDI and integrin β3 and their mitochondrial oxidoreductive activity was determined at 24 h.p.i. using the resazurin reduction test. Most of the antibodies tested at two different concentrations (4.0 or 0.4 μg/mL) did not significantly affect cell viability. However, a slight decrease in cell viability was observed when cell pre-treatment was performed with antibodies against Hsp90, Hsc70 or PDI at the highest concentration used (4 μg/mL) (Figure 6E). Non-infected cells were used to normalize for 100% cell viability, while Wt1-5-infected cells at MOI 2 were used as a negative control of cell viability. A non-related isotype antibody against potato virus Y was also used as a control (data not shown). Cell viability determined with the trypan blue exclusion test showed a profile similar to that found with the resazurin reduction test (Figure 6F). Since the antibodies used react with cell surface proteins, it appears to be plausible to suggest that the cellular proteins studied are involved in virus entry, without excluding other antibody-induced intracellular effects that might contribute to a successful virus life cycle.

### 3.7. Colocalization of HSPs, PDI and β3 and Wt1-5 Structural Antigens

**Figure 7:**
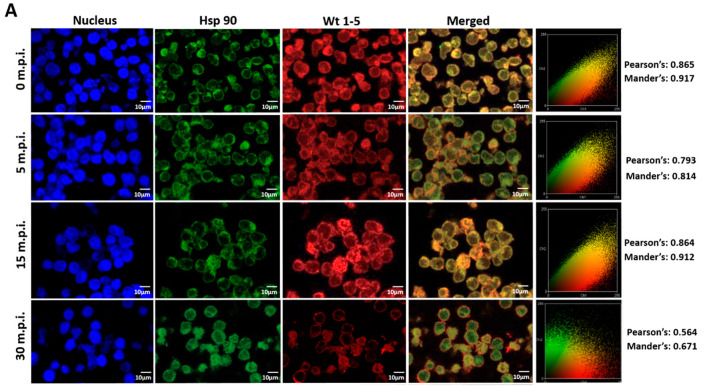

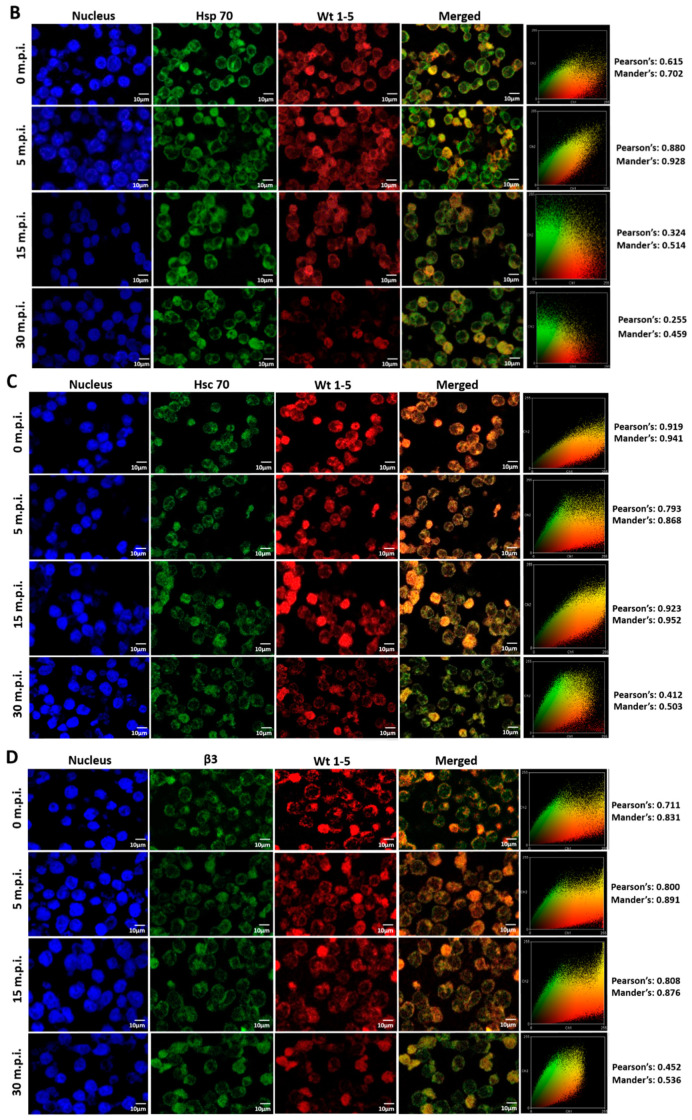

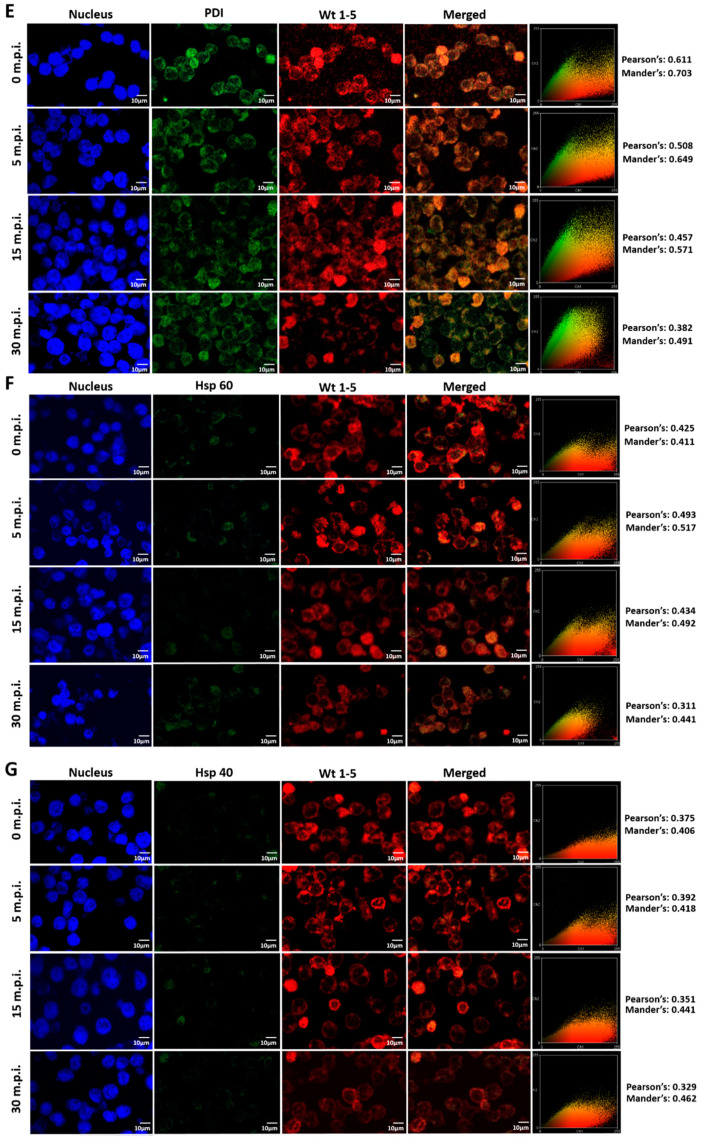
Colocalization of cellular proteins and Wt1-5 structural proteins. Reh cells that had been incubated for 15 min at 4 °C were inoculated with trypsin-activated Wt1-5 and collected at 0, 5, 15 and 30 min post-infection (m.p.i.) to be fixed with 4% PFD. Cellular proteins were detected with antibodies labeled with Alexa Fluor 488 (green), Wt1-5 structural proteins with Alexa Fluor 568 (red) and nuclei with DAPI (blue). Colocalization was analyzed using a global statistical approach that executes intensity correlation coefficient-based (ICCB) analyses and determining Pearson’s correlation coefficient and Mander’s correlation coefficient for green and red channels. Pearson’s and Mander’s indexes for each colocalization analyzed are shown on the right of each panel. Representative photographs of colocalization analysis for Hsp90 (**A**), Hsp70 (**B**), Hsc70 (**C**), integrin β3 (**D**), PDI (**E**), Hsp60 (**F**) and Hsp40 (**G**) are shown. Images were obtained using a Nikon C1 confocal laser scanning microscope controlled by Nikon’s EZ-C1 software ver. Gold. 3.90 with Objective Plan. Apo. 100x/NA1.40/WD0.13 Oil PFS. Images were analyzed with the ImageJ 1.44p Java 1.6.0-20(32-bit) software. The results shown are representative of two different assays.

Since rotavirus has been reported to use cell surface proteins from MA104 cells, such as Hsc70, PDI and integrin β3, we wanted to determine whether Wt1-5 colocalizes at least with the cellular proteins Hsp90, Hsp70, Hsp60, Hsp40, Hsc70, PDI and integrin β3 from Reh cells. To test for a possible colocalization, cells were inoculated with Wt1-5 at MOI 6 as indicated above. Infected cells were harvested at 0, 5, 15 and 30 min after inoculation, and then, fixed with PFD. The indicated cellular proteins were reacted with the corresponding antibodies and detected with Alexa Fluor 488-labeled antibodies (green), while the Wt1-5 structural proteins were detected with Alexa Fluor 568-labeled antibodies (red) and nuclei with DAPI (blue). Cells were analyzed by confocal microscopy using a global statistic approach that executes intensity correlation coefficient-based (ICCB) analyses and determining Pearson’s correlation coefficient and Mander’s correlation coefficient for green and red channels (Figure 7A–G). After this analysis, colocalization was found between Wt1-5 structural proteins and Hsp90 (Figure 7A), Hsp70 (Figure 7B), Hc70 (Figure 7C) and integrin β3 (Figure 7D) at 0, 5 and 15 min after inoculation (Pearson’s index from 0.793 to 0.928 and Mander’s index from 0.814 to 0.952). This correlation decreased after 30 min post-inoculation. Colocalization of viral structural proteins and PDI showed non-specific correlation indexes (Pearson’s index from 0.382 to 0.611 and Mander’s index from 0.491 to 0.703) (Figure 7E). Colocalization analysis for Hsp60 (Figure 7F) and Hsp40 (Figure 7G) showed non-correlation (Pearson’s index from 0.311 to 0.493 and Mander’s index from 0.406 to 0.517).

## 4. Discussion

The interaction between tumor cell-adapted rotaviruses and their host cell that ends in cell death is not a well-understood process in the context of their potential use as OVs. Virus-infected cells can undergo apoptosis as a protective response that limits virus infection. Although the mechanism supporting apoptosis induction by rotavirus is not well characterized, there are reports suggesting that rotavirus regulates apoptosis during infection of cultured cell lines [85,86,87]. Therefore, we investigated the effect of rotavirus Wt1-5 infection on the expression of some markers of apoptotic cell death and its relationship with the expression of Hsp90, Hsp70, HSp60, Hsp60, Hsp40, Hsc70, integrin β3 and PDI in the cytoplasmic cell membrane. Our results showed that Reh cells were certainly infected by Wt1-5 in terms of the growing accumulation of viral antigens and infectivity, while PBMCs were not. It appears to be that the very low expression of cell surface HSPs in PBMCs was not enough to make possible the infection by Wt1-5. In addition, Wt1-5 infection of Reh cells induced cell mortality as evidenced by a decreased cell viability that was nearly proportional to the MOI values used. This effect was not detected in PBMC. The use of relatively lower MOI values, such as those below 3, together with post-infection times longer than 12 h, suggests that some of the effects of viral infection on apoptotic markers have to be seen in the context of a multistep growth curve, in which spreading of infectious virions to other cells can occur in a non-synchronous way. Additionally, rotavirus Wt1-5 was able to selectively infect Reh cells but not PBMCs

The ability of Wt1-5 infection to induce the expression of caspases 3 and 8 and pro-apoptotic proteins BAX, Bcl2, and BID suggest that virus infection, at least at late stages of the viral life cycle, leads to apoptotic cell death. However, our experimental design does not allow us to differentiate if the virus-induced expression of these apoptotic markers takes place through direct or indirect interaction of viral proteins with the induced proteins. The virus infection-associated induction of the apoptotic markers studied is also suggested by the fact that most of the cells showing viral antigens were also positive for increased levels of these apoptotic markers, and that the proportion of cells being positive to increased accumulation of these markers was increased with increasing MOI values. Moreover, the PARP-1 cleavage, TUNEL and agarose gel electrophoresis assays showed that Wt1-5 infection was an inducer of DNA fragmentation, a key feature of apoptotic cell death. Our analysis of the expression of cell death markers by Western blotting showed that the amount of Bcl-2 decreased in Wt1-5-infected cells at 24 h.p.i. This result was found to be significant when compared to the non-infected cells at the same post-infection time. In contrast, the total amount of Bax remained the same in the infected cells, except its active form, which was increased. Thus, the Wt1-5 infection increased the ratio of Bax/Bcl-2 at 24 h.p.i. Bax is known to be an activating molecule leading to cytochrome C release, which in turn, leads to activation of the effector caspase 3 [88]. The increase in active Bax may just contribute to the induction of the mitochondrial apoptotic pathway, as previously reported [89]. In fact, the inhibition of the pro-survival function of Bcl-2 has been shown to be essential for Bax activation, since Bcl-2 can compete with Bax for cytochrome C release [90,91]. It sounds plausible to suggest that the Wt1-5 infection can contribute to Bax activation through the decrease in the Bax/Bcl-2 complex number in the mitochondrial membrane. It has been previously reported that non-structural rotavirus proteins NSP4 and NSP6 are able to affect the mitochondrial function, resulting in the dissipation of mitochondrial membrane potential during rotavirus infection [86,92]. Interestingly, mitochondrial Hsp60 mediates the NSP4 translocation to mitochondria, leading to the dissipation of mitochondrial membrane potential during the rotavirus infection [93].

Although we analyzed the expression of the total BID rather than its active form (tBID), it is well known that proteins with BH3 domain, such as BID, can suppress Bcl-2 activity to inhibit apoptosis by interacting with Bcl-2 to later activate Bax [94]. In that way, BID can be activated by caspase 8 resulting in Bax activation and release of cytochrome C [95]. An interesting finding is that inoculation with UV-inactivated Wt1-5 produced an increase in total BID concentration. It has been reported that exposure of monolayers of MA104, HT-29 and Caco-2 cells to inactivated rotavirus or dsRNA from rotavirus produces changes in the integrity of monolayers, affectation of metabolic activity and late reduction in cell viability [96]. These antecedents may explain why the increase in total BID expression in cells exposed to UV-inactivated Wt1-5 did not lead to an increase in active Bax concentration, caspase-3, and cytochrome C. Previous data reported by others suggest that most childhood ALL cell lines (RCH, Reh, SUP B15, and HAL01) rarely have mutations in p53 in the case of B-cell precursors, although they may be more frequent in Burkitt lymphoma [97,98,99]. Although we did not assess whether the Reh cell line used has the wild-type p53 version, the presence of this version in the Reh cell line would give support to the hypothesis that the p53-dependent cell cycle arrest and apoptosis via the intrinsic pathway is favored by rotavirus infection, as it has been reported for other cases [85,89].

Cell membrane permeability changes caused by Wt1-5 infection can also suggest a relationship between virus infection and apoptotic cell death, as apoptosis has been related with a series of physical changes occurring in the cell membrane which include the increase in permeability to some dyes [100,101]. The dyes used are not specific for characterizing apoptosis and they should be used in conjunction with other methods for apoptosis detection. The mitochondrial membrane potential (∆Ψm) changes observed during Wt1-5 infection suggest that this rotavirus isolate can trigger an early and key event of apoptosis, since when the mitochondrial membrane becomes permeabilized, it releases pro-apoptotic factors into the cytosol [86,89,92,93].

Simian rhesus rotavirus strain RRV has been reported to induce caspase 3 activation, DNA fragmentation, and cleavage of PARP-1 in both MA104 and Caco-2 cells [85]. In addition, RRV induced the release of cytochrome C from mitochondria into the cytosol, suggesting that an apoptotic pathway is activated [89]. Rotaviral enterotoxin non-structural protein 4 (NSP4) has been shown to target mitochondria for inducing apoptosis. The authors of this study showed that NSP4 exerts its apoptotic effects by interacting with mitochondrial membrane proteins adenine nucleotide translocator and voltage-dependent anion channel (VDAC), leading to dissipation of mitochondrial potential, release of cytochrome C from mitochondria, and caspase activation. The rotavirus non-structural protein 4 (NSP4) can translocate to mitochondria, causing the dissipation of the mitochondrial membrane potential and inducing apoptosis during the early infection [86,102]. Moreover, the rotavirus infection increases the concentration of free Bax, contributing in this way, to the mitochondrial apoptotic pathway through caspase 3 activation, DNA fragmentation, and poly (ADP-ribose) polymerase cleavage [89]. On the other hand, rotavirus NSP1 counteracts the virus infection-induced apoptosis to facilitate the viral cycle during the early steps of infection [103,104]. In this way, NSP1 counteracts the pro-apoptotic signaling of NSP4. Our results indicate that apoptosis could be a mechanism by which rotavirus Wt1-5 kills Reh cells. Though, the detailed molecular mechanisms by which Wt1-5 triggers the events leading to apoptotic cell death remain to be elucidated. However, more studies are needed to discard the possibility that Wt1-5 could infect non-tumor cells.

Once the percentage of cells being positive to HSPs (Hsp90, Hsp70, Hsp60, Hsp40, Hsc70), integrin β3, and PDI and also the intensity of their expression were established, the pre-treatment of Reh cells with antibodies against these cellular proteins resulted in differential infection rates and numbers of infectious particles produced. However, pre-treatment of Reh cells with antibodies to Hsp60 and Hsp40 did not affect neither the infection rate nor virus yield. These results allow us to suggest that the cellular proteins affecting virus infectivity after antibody pre-treatment are involved in the early steps of Wt1-5 entry, although until now, the detailed mechanisms of their involvement are not clear. It is worth highlighting that a combined pre-treatment with antibodies to HSPs did not have an additive inhibitory effect. The pre-treatment of cells with a non-related isotype antibody did not affect infectivity, suggesting that a steric hindrance is not responsible for the inhibitory effect. Pre-treatment of cells with antibodies to some cell surface proteins to block infection has been well documented for several viruses [105] including Zika virus (ZIKV) [106] and Japanese encephalitis virus (JEV) [107,108], which use cell surface Hsp70 and Hsp90β, respectively. Cell surface Hsc70 has been reported as one of the receptors used by rotavirus during entry into MA104 cells [51].

Likewise, polyclonal anti-Hsp70 antibodies blocked the entry of JEV into Neuro2a mouse neuroblastoma cells, suggesting that Hsp70 serves as a putative receptor for JEV in Neuro2A cells and in human hepatoma cells (Huh7). The recruitment of Hsp70 in lipid rafts was required for activation of the PI3K/Akt signaling pathway during the early steps of JEV infection [109]. Antibodies to Hsc70 have also been implicated in the blockade of Chikungunya virus entry into C6/36 Aedes albopictus cells. [110]. On the other hand, intracellular Hsp90 has been reported to positively regulate rotavirus replication by modulating the activation of the virus-induced PI3K/Akt and NF-kB pathways through the active association of Hsp90 with the non-structural protein 3 of rotavirus (NSP3) [111,112]. Furthermore, other authors have found that rotavirus infection induces the intracellular expression of Hsp70 in response to viral infection, in a transient and rotavirus strain-dependent manner [113]. Rotavirus has been shown to use different types of endocytosis for entry into the target cell. In addition, rotaviruses can bind to other cell surface molecules, such as integrins α2β1, αXβ2, and ganglioside with attached sialic acid [114,115,116], which were not analyzed in the present study. Probably, these cellular proteins play a role during the early steps of rotavirus entry into the Reh cells, as an early colocalization between viral structural proteins and some of the cellular proteins studied was observed. However, these cellular proteins may play other roles than simply facilitating virus entry. It cannot be excluded that these cellular proteins induce intracellular mechanisms that are needed for a successful viral life cycle. The current understanding of the mechanisms used by rotavirus to enter cells explains their natural and selective tropism for enterocytes. Rotavirus entry into the cells is a multistep process involving an initial binding to sialic acid (SA) [117] followed by interactions with some integrins, Hsc70 and PDI [51,54] in an order not yet well established. Alternative entry pathways using different receptors and co-receptors have been suggested [114]. Here, we show the tropism and selectivity exhibited by Wt1-5 for Reh cells, a human acute lymphoblastic leukemia cell line, that have a higher expression of the cell surface Hsp90, Hsp70, Hsc70, PDI and αVβ3 as compared with their very low or absent expression in PBMCs. This suggests that the potential of Wt1-5 for specific targeting of tumor cells lies in the over-expression of HSPs on the cell surface.

Overall, the present results support the possibility of conducting further research about the use of rotaviruses as potential oncolytic agents by assaying them in isolated human acute lymphoblastic leukemia cells and also in animal models aimed at determining their efficacy and safety.

## 5. Conclusions

The present results allow us to conclude that rotavirus Wt1-5 can be a potential candidate to be further studied as an oncolytic agent that can use cell surface proteins Hsp90, Hsp70, Hsc70, PDI, and integrin β3 to infect human acute lymphoblastic leukemia cells expressing these cellular proteins. Furthermore, the cytotoxic effects induced by Wt1-5 infection are compatible with an apoptosis-induced cell death which is dependent on the MOI and post-infection times.
